# *Pneumocystis* pneumonia after use of corticosteroids in a man with severe alcoholic hepatitis: A case report

**DOI:** 10.1097/MD.0000000000018696

**Published:** 2020-01-10

**Authors:** Min Woo Chung, Uh Jin Kim, Chung Hwan Jun, Sung Bum Cho, Seon Young Park, Chang Hwan Park, Hyun Soo Kim, Sung Kyu Choi, Jong Sun Rew

**Affiliations:** a Department of Gastroenterology and Hepatology; b Department of Infectious diseases, Chonnam National University Hospital and College of Medicine, Gwangju, South Korea.

**Keywords:** alcoholic hepatitis, corticosteroids, glucocorticoids, hepatotoxicity, *Pneumocystis* pneumonia, trimethoprim-sulfamethoxazole

## Abstract

**Rationale:**

Severe alcoholic hepatitis (AH) has a very high mortality rate. Current guidelines recommend oral corticosteroids as first-line agents in individuals with severe AH to reduce short-term mortality. However, systemic corticosteroids have serious adverse effects. In individuals with AH, infection, which is one of the complications of steroid use, can result in serious outcomes, such as acute-on-chronic liver failure. Pneumocystis pneumonia (PCP) is a life-threatening opportunistic infection which may occur when high-dose corticosteroids are prescribed for more than 1 month. Therefore, when high-dose corticosteroids are used, providing PCP prophylaxis is warranted. Although trimethoprim-sulfamethoxazole (TMP-SMX) is the drug of choice for the prophylaxis of PCP, its hepatotoxicity limits its use in patients with severe AH who are on high-dose corticosteroids. Moreover, there is a lack of consensus on which drugs should be used for PCP prophylaxis in individuals with severe AH who are on glucocorticoid treatment. Herein, we report a case of a 43-year-old male with fatal PCP that occurred after the use of corticosteroids for severe AH.

**Patient concerns:**

A 43-year-old alcoholic man presented with a hematoma on his right leg. His liver function was poor, and he was he was diagnosed with severe AH and treated with oral corticosteroids for 26 days. After glucocorticoid treatment, he developed a productive cough.

**Diagnoses:**

A sputum PCR test was positive for *Pneumocystis jirovecii*.

**Interventions:**

He was initially treated with TMP-SMX and required artificial ventilation.

**Outcomes:**

He developed disseminated intravascular coagulation and multi-organ failure, and died 10 days after starting TMP-SMX.

**Lessons:**

To date, prevention of PCP in individuals with severe AH who are on corticosteroids has been overlooked. This case illustrates the need for prophylaxis of PCP in individuals with severe AH taking corticosteroids.

## 1 Introduction

Alcohol-related liver disease is a lethal disease, with >5% mortality. Alcoholic hepatitis (AH) is a clinical syndrome of jaundice, with or without other signs of liver decompensation, in chronic alcohol abusers. The 3-month mortality rate of patients with severe AH is as high as 50%.^[1]^ Corticosteroids are widely used to treat individuals with severe AH to reduce short-term mortality.^[2]^ However, systemic corticosteroids have multiple adverse effects on the immune system, bone and muscle, and metabolic and hormonal effects. Opportunistic infections in particular, can result in serious outcomes including *Pneumocystis* pneumonia (PCP). PCP is a life-threatening infection that occurs in immunocompromised patients. In 1 retrospective study, patients without HIV infection who were treated with ≥16 mg of prednisone for 8 weeks had a significantly increased the risk of PCP.^[3]^ Therefore, when high-dose corticosteroids are used, PCP prophylaxis is warranted.^[4]^ Although trimethoprim-sulfamethoxazole (TMP-SMX) is the drug of choice for prophylaxis of PCP,^[5]^ its hepatotoxicity limits its use in individuals with severe AH who are on high-dose corticosteroids. Medical guidelines do not mention PCP prophylaxis in individuals with severe AH who are on corticosteroids. Herein, we report a fatal case of PCP in a 43-year-old man who was taking corticosteroids for severe AH and was treated with TMP-SMX. Patient has provided informed consent for publication of the case.

## 2 Case report

A 43-year-old man presented to the emergency center of a tertiary hospital with pain of his right lower leg. He had a 20-year history of heavy alcohol use and had been diagnosed with alcoholic liver cirrhosis 2 months previously. Three days before admission, he had bumped his right leg against a table. His leg had become swollen and painful, and he had developed a hematoma. He visited a local clinic, where laboratory examination revealed a hemoglobin (Hgb) of 4.2 g/dL. After transfusion of 7 units of red blood cells, he was transferred to our hospital for further evaluation and management.

**Figure 1:**
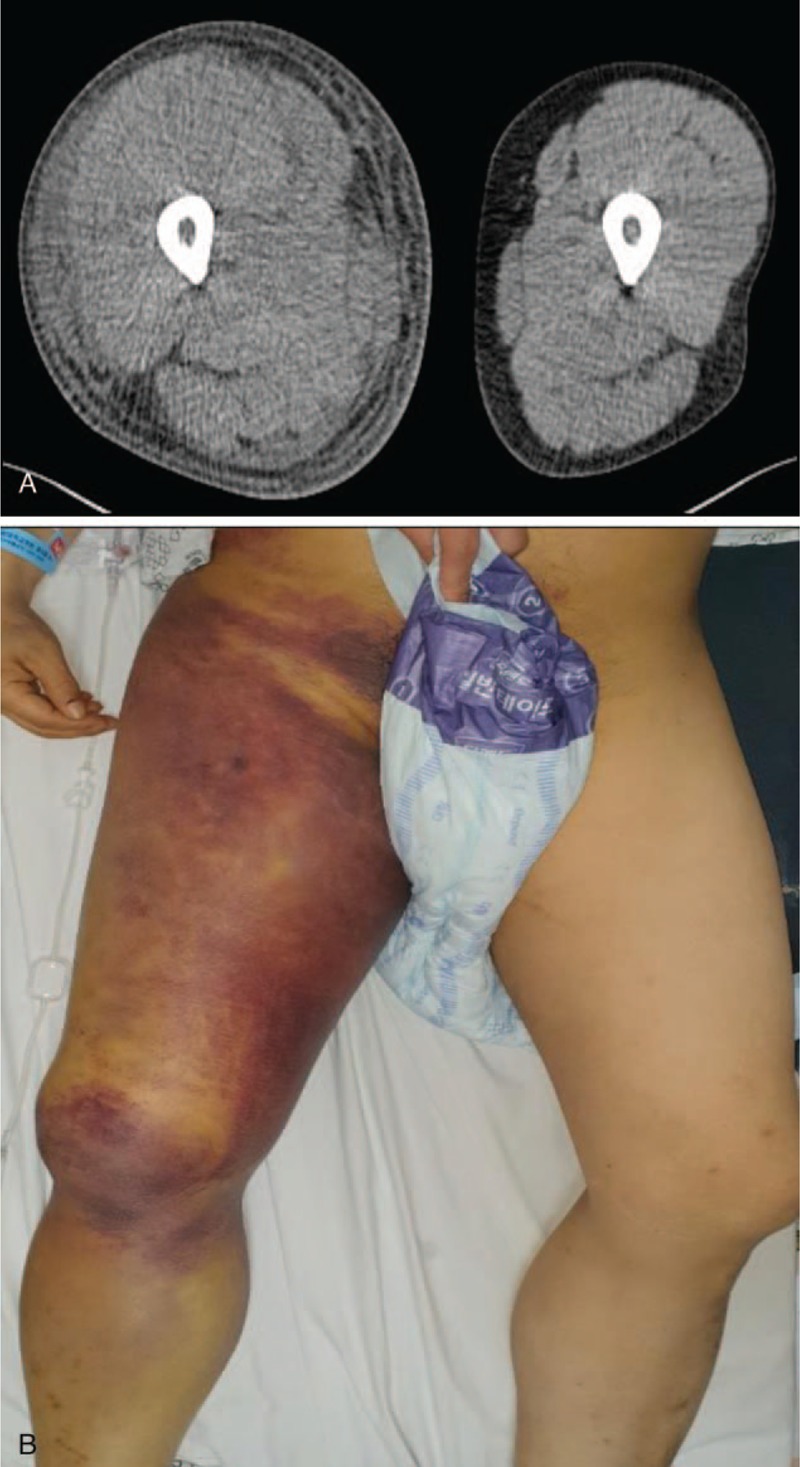
Computed tomography scan showing soft tissue swelling with some hematoma in right leg (A); and gross appearance of intramuscular hematoma of right thigh (B) on the day of admission.

On admission, his Hgb level had increased to 7.8 g/dL. Other tests revealed an aspartate transaminase (AST) of 145 IU/L, an alanine aminotransferase (ALT) of 38 IU/L, a total bilirubin 8.7 mg/dL, an albumin of 3.1 g/dL, and a prothrombin time international normalized ratio (PT-INR) of 1.78. His Child-Pugh score was 9 points, and his Model for End-stage Liver Disease (MELD) score 21, both of which indicated severe liver disease. A computed tomography (CT) scan revealed a large hematoma on his right leg (Fig. 1), which explained the swelling and anemia. His calculated Maddrey Discriminant Function (mDF) score for AH was 42, which indicated a poor prognosis.

**Figure 2:**
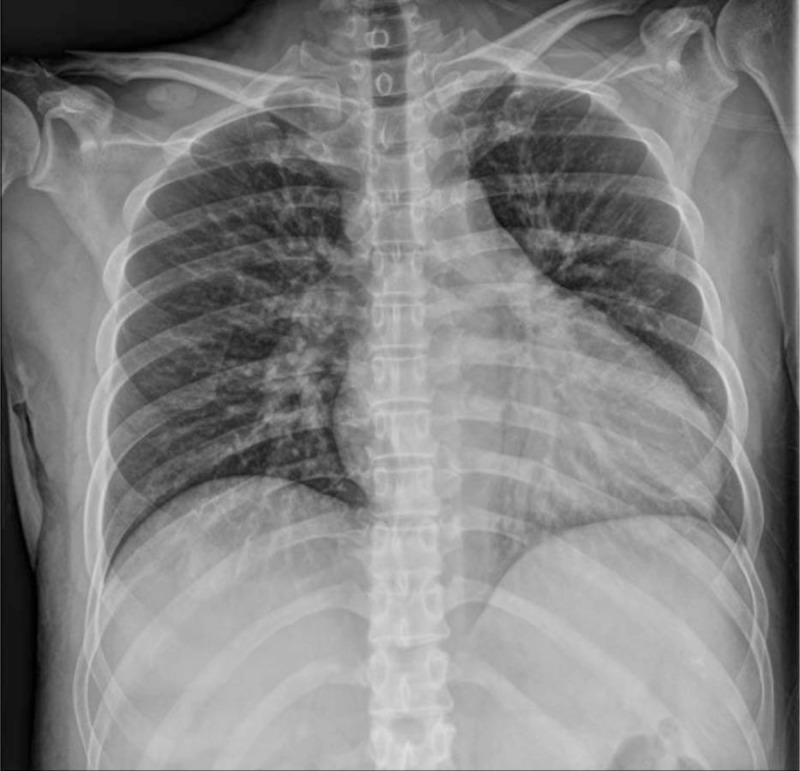
Chest X ray (Antero-posterior scan) on the day of admission, showing no active lung lesion.

He was treated with methylprednisolone, 40 mg daily, thiamine, and vitamin B1. He had no respiratory symptoms and there were no active lung lesions on chest X-ray (Fig. 2). One week after starting corticosteroids, his Lille score was 0.22, and his bilirubin had decreased, which indicated that his AH had responded to the corticosteroids. Therefore, we continued treatment with corticosteroids.

Nine days after admission, he developed fever and urinary frequency. Urinalysis revealed urine nitrite and pyuria and *Proteus mirabilis* was identified in his urine culture, and so his was provided with ciprofloxacin for 3 days. We continued methylprednisolone 40 mg for 14 days, and then we decreased the dose by 10 mg/day every 4 days, and discontinued the methylprednisolone after 26 days. Twenty-six days after admission, his laboratory findings included an AST of 75 IU/L, an ALT of 47 IU/L, a total bilirubin of 12.39 mg/dL, an albumin of 3.1 g/dL, and a prothrombin time international normalized ratio (PT-INR) of 1.52. He developed a fever, and so ciprofloxacin was prescribed empirically. His fever persisted, and 28 days after admission, he developed hemoptysis, a productive cough, and exertional dyspnea.

**Figure 3:**
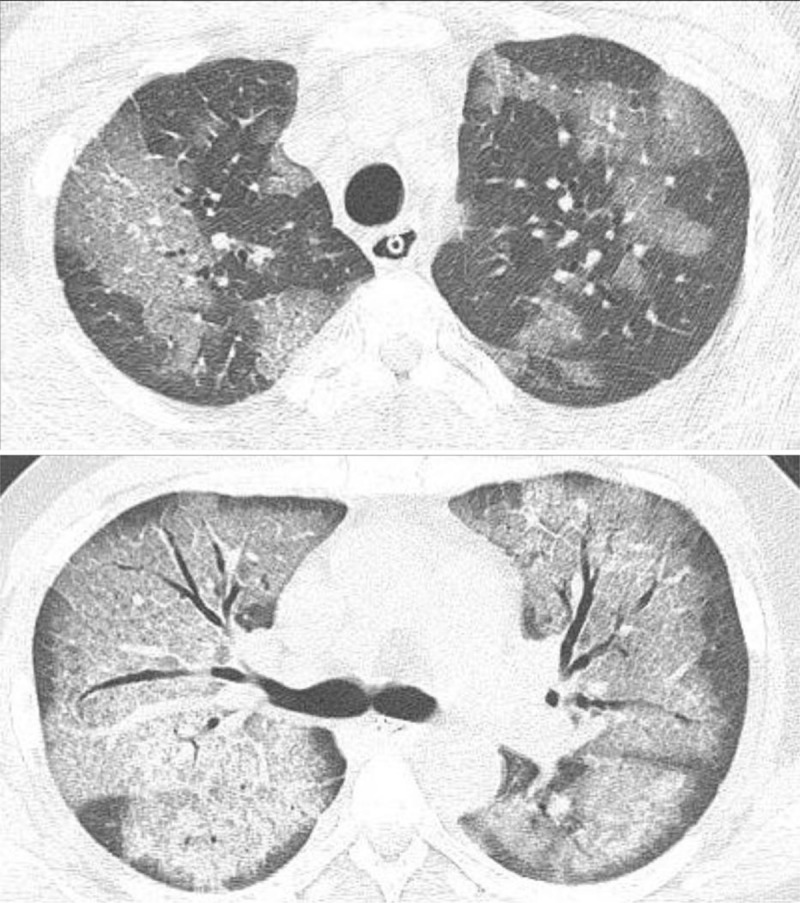
Computed tomography scan performed 28 days after admission, showing diffuse/patchy ground glass opacities and consolidations with interlobular septal thickening in both lungs.

A chest CT scan revealed diffuse patchy ground-glass opacities and consolidations, with interlobular septal thickening in both lungs (Fig. 3). *Pneumocystis jirovecii* polymerase chain reaction (PCR), acid-fast bacilli (AFB) staining, TB culture, and bacterial and fungal culture of sputum were performed. We suspected that he had hospital-acquired pneumonia, and so we changed the ciprofloxacin to piperacillin/tazobactam and levofloxacin. We also prescribed TMP-SMX and restarted corticosteroids.

**Figure 4:**
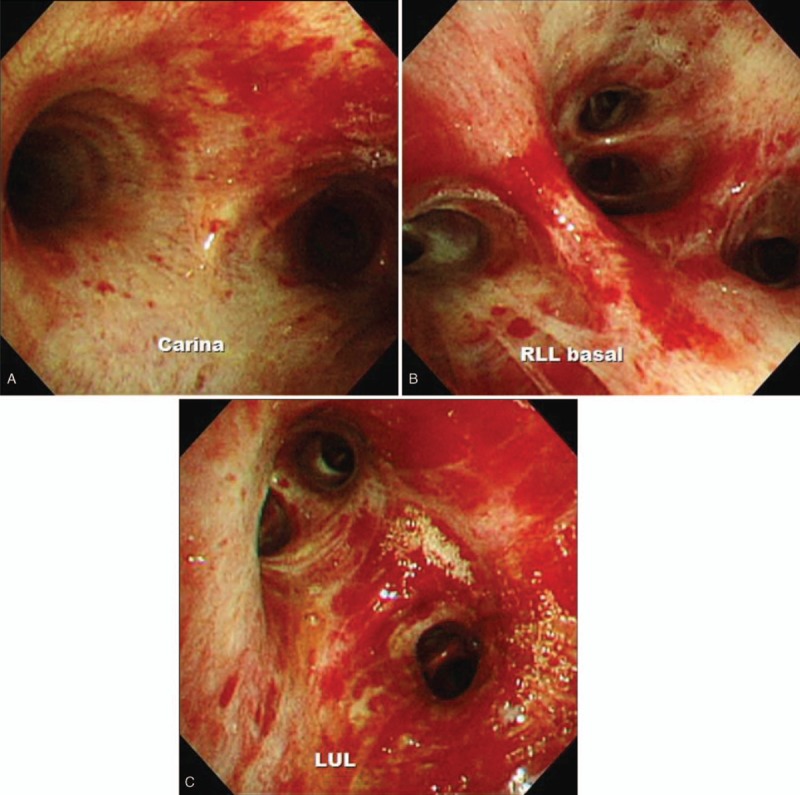
Bronchoscopy performed 30 days after admission, showing diffuse alveolar hemorrhage.

Two days later he became hypoxic, and so he was intubated and started on mechanical ventilation. During the intubation he had massive hemoptysis and diagnostic bronchoscopy revealed diffuse alveolar hemorrhage (Fig. 4). Bronchoalveolar lavage (BAL) specimens were sent for culture, and PCR testing for respiratory viruses, (including influenza, adenovirus, and rhinovirus), His PCR tests were negative for *Pneumocystis jirovecii* and *Mycobacterium tuberculosis* complex. Cytology of a BAL sample revealed benign atypia. However, a PCR test done a few days earlier had been positive for *Pneumocystis jirovecii*. We were already using TMP-SMX because we suspected that he had PCP, and we increased the dose of corticosteroids because of his acute respiratory distress syndrome.

Despite intensive medical treatment, he developed disseminated intravascular coagulation and progressive multi-organ failure, and died on 10 days after starting TMP-SMX, 39 days after admission.

## 3 Discussion

This case report describes a patient with severe AH who died from PCP after treatment with corticosteroids. To date, prevention of PCP in patients with severe AH who are on corticosteroid treatment has been overlooked, and this case emphasizes the need for prophylaxis of PCP in patients with severe AH who are on corticosteroids.

PCP is an opportunistic infection that occurs when the immune system is compromised, as is seen in patients with acquired immunodeficiency. In a United States cohort of individuals with human immunodeficiency virus (HIV), the incidence of PCP was 3.9 per 1000 person-years between 2003 and 2007, a dramatic decrease from 29.9 per 1000 person-years between 1994 and 1997.^[6]^ Our patient's sputum tested positive result for *Pneumocystis jirovecii* on PCR. To make a definite diagnosis of PCP, tinctorial (dye-based) staining, fluorescent antibody staining, or PCR-based testing of respiratory specimens should be performed. In patients without acquired immunodeficiency syndrome, quantitative PCR for the diagnosis of PCP has not yet been established, but it can be of particular use in patients without HIV infection, because the sensitivity of microscopy with staining is lower than in HIV-infected patients.^[7]^

Corticosteroids have been used as the first-line agent in the treatment of severe AH.^[8]^ Prednisolone 40 mg/day, or methylprednisolone 32 mg daily, intravenously is recommended in patients with an mDF score of ≥32.^[9]^ However, the efficacy of corticosteroids has not yet been determined. In previous clinical trials, including the STOPAH trial,^[10]^ participants in the corticosteroids groups have had a lower mortality rate than those in the placebo group at 28 days.^[11]^ In contrast, previous a meta-analysis found that mortality was not significantly different from the placebo group.^[12]^ Moreover, the adverse effects of treatment with corticosteroids in severe AH have not been researched. Discontinuation of corticosteroids is recommended in the absence of treatment response (defined as Lille score ≥0.45) after one week of treatment, because the risk of side effects is believed to be greater than the benefit.^[13]^

Infection is a common and serious complication of severe AH. According to recent meta-analysis, the cumulative incidence of infection is 20% after 28 days, increasing to 65% after 3 months.^[14]^ The cause of high infection rate is mainly related to the presence of basal cirrhosis, which is related to bacterial overgrowth, dysbiosis, and impaired immunity.^[15]^ Bacterial infection in individuals with AH is difficult to differentiate from noninfectious inflammation, but 25% of patients hospitalized due to severe AH develop bacterial infections after steroid treatment.^[16]^ Although bacterial infections account for the highest percentage of infections, invasive aspergillosis and PCP is a clinically significant proportion and are associated with a poor prognosis. Studies have shown that 8% of patients with severe AH develop PCP, and that prognosis is particularly poor.^[17]^ A 2014 Cochrane review of PCP prophylaxis in immunocompromised patients without HIV infection, found that PCP prophylaxis is warranted when the risk of PCP is higher than 6%.^[4]^ Compared to no treatment, there was an 85% reduction in the occurrence of PCP in patients receiving prophylaxis with TMP-SMX, and PCP-related mortality was significantly reduced, with relative risk of 0.17. For this reason, PCP prophylaxis is warranted in patients taking >20 mg/day of corticosteroids for more than 1 month.^[3]^ Although a dose >20 mg/day of corticosteroids is usually used to treat severe AH, even when the risk of PCP is >6%,^[17]^ prophylaxis for PCP is often not provided because the best prophylactic drug, TMP-SMX, is hepatotoxic. Alternative preventive regimens include atovaquone, dapsone, with or without pyrimethamine, and aerosolized pentamidine.^[18]^ However, none of these agents have been compared with TMP-SMX in a randomized trial, and oral drugs such as TMP-SMX have relatively higher hepatotoxicity. Atovaquone undergoes enterohepatic recirculation, and dapsone is metabolized in the liver (acetylation and hydroxylation).^[18]^ Pentamidine isethionate is active against *Pneumocystis* and several protozoal pathogens. When using aerosolized pentamidine, locoregional symptoms, such as sore throat, oral paresthesia, a metallic taste, cough, and bronchospasm are major side effects, and knowledge of its hepatotoxicity is limited. Pentamidine can be used at a dose of 300 mg in a Respirgard II nebulizer once a month.^[19]^ Although patients treated with aerosolized pentamidine have a significantly lower survival rate than patients treated with other regimens,^[20]^ several guidelines recommend aerosolized pentamidine as an alternative regimen.

In conclusion, therapy with corticosteroids is recommended for severe AH. Special attention should be paid to side-effects when using high-dose corticosteroids for treatment. The incidence of PCP in individuals with severe AH who are treated with corticosteroids is thought to be >8%,^[17]^ and PCP is usually fatal. Appropriate monitoring and prevention of PCP is needed. If TMP-SMX, known as the best drug for prophylaxis for PCP, cannot be used due to hepatotoxicity, an alternative means of prophylaxis should be chosen. Aerosolized pentamidine may be a good alternative when considering hepatotoxicity, but further study is needed on its use for prophylaxis of PCP in individuals with severe AH.

## Author contributions

**Conceptualization:** Chung Hwan Jun.

**Investigation:** Uh Jin Kim.

**Project administration:** Sung Bum Cho.

**Resources:** Seon Young Park.

**Supervision:** Jong Sun Rew.

**Visualization:** Chang Hwan Park.

**Writing – original draft:** Min Woo Chung.

**Writing – review & editing:** Hyun Soo Kim, Sung Kyu Choi.

Min Woo Chung orcid: 0000-0003-3144-1450.
